# Sex and age differences in regional distribution of bone mineral density in the pubic symphysis using Computed Tomography Osteoabsorptiometry (CT-OAM)

**DOI:** 10.1186/s12891-026-09566-7

**Published:** 2026-02-07

**Authors:** Amélie Poilliot, Niels Hammer, Magdalena Müller-Gerbl

**Affiliations:** 1 Anatomical Department of Biomedicine, Musculoskeletal Research, University of Basel, Pestalozzistrasse 20 , Basel, 4056 Switzerland; 2 Division of Macroscopic and Clinical Anatomy, Gottfried Schatz Research Center, Medical University of Graz, Auenbruggerplatz 25, Graz, Austria; 3 Department of Orthopedic and Trauma Surgery, University of Leipzig, Leipzig, Germany; 4 Division of Biomechatronics, Fraunhofer Institute for Forming Tools, Dresden, Germany

**Keywords:** Computed tomography osteoabsorptiometry, Hounsfield units, Subchondral bone

## Abstract

**Background:**

Subchondral bone (SCB) remodels in response to long-term mechanical loading, with mineralisation patterns reflecting chronic mechanical stress. Computed tomography osteoabsorptiometry (CT-OAM) enables non-invasive visualisation of these adaptations. While CT-OAM has been widely applied to several joints, its use in the pubic symphysis is limited. This study aimed to identify sex- and age-related differences in mineralisation patterns and quantify bone mineral density (BMD) distribution across the symphyseal surfaces.

**Methods:**

CT scans from 85 individuals (51 males, 34 females; age range 18–97 years) were analysed, generating 170 symphyseal surfaces. Segmented three-dimensional reconstructions were processed into HU-based densitograms. Mineralisation was classified qualitatively into three patterns: diffuse across the surface (Pattern 1), ventral border with/without inferior apex involvement (Pattern 2), and dorsal border with/without inferior apex involvement (Pattern 3). Quantitatively, each surface was subdivided into six anatomical subregions, and mean HU values were compared by sex, side, and age.

**Results:**

Across all specimens, Pattern 2 predominated (58%), with Pattern 2 most frequent in males (76%) and Pattern 3 in females (55%). High bilateral conformity (81%) was observed. Males exhibited significantly higher mean HU values than females (554 ± 180 HU vs. 374 ± 111 HU, *p* < 0.01), with greater BMD across all subregions. Region-specific analyses revealed highest mineralisation anteriorly and inferiorly in males, while females displayed increased posterior mineralisation. No significant correlation was found between overall BMD and age; however, females demonstrated a weak negative correlation in the ventral middle region (*r* = − 0.24, *p* < 0.05).

**Conclusion:**

This study provides the first systematic CT-OAM analysis of pubic symphyseal SCB mineralisation. Findings highlight sex-specific patterns, with males demonstrating greater anterior and inferior mineralisation, and females exhibiting posterior dominance. Males also displayed higher overall BMD, reflecting greater chronic loading. These results deepen understanding of pelvic biomechanics and may inform future research on conditions such as osteitis pubis.

**Supplementary Information:**

The online version contains supplementary material available at 10.1186/s12891-026-09566-7.

## Introduction

Subchondral bone (SCB) adapts to repetitive mechanical loading by altering its mineral density, a process detectable via computed tomography (CT) osteoabsorptiometry (OAM) [1–4]. This adaptation aligns with Wolff’s law and Frost’s Mechanostat principle which describe how bone remodels in accordance with the magnitude and direction of mechanical stresses it experiences [1, 3, 5, 6]. CT-OAM offers a non-invasive method to visualize Hounsfield Unit (HU)-based densitograms derived from standard CT scans, enabling the spatial assessment of mineral distribution beneath articular surfaces [1, 2, 4, 7–10]. These densitograms represent the distribution of subchondral mineralisation across the surface displayed as a HU-based colour maps. The principle that “morphology reveals biomechanics” underpins this approach, allowing long-term joint loading patterns to be inferred from subchondral mineralisation maps.

CT-OAM has been applied extensively to synovial joints such as the glenohumeral, hip, knee, ankle, and sacroiliac joints, where it has proven useful in identifying region-specific stress adaptation and correlating mineralisation with the mechanical competence of the SCB [1–5, 7–10]. In contrast, the pubic symphysis—a fibrocartilaginous joint that plays a critical role in load transfer across the anterior pelvic ring—has received comparatively little attention. This is notable given its exposure to complex combinations of compressive, tensile, and shear forces during daily activities and locomotion.

Only a limited number of studies have applied CT-based methods to the pubic symphysis [1, 4, 11]. Using CT imaging, Putz, et al. [12] conducted one of the first quantitative assessments, revealing sex- and region-specific differences in subchondral mineralisation, describing higher anterior density in males and increased posterior density in females. Subsequent studies have suggested age-related increases in porosity and alterations in symphyseal morphology, potentially reflecting declining mechanical integrity with advancing age [13]. Additional morphometric work combining CT and magnetic resonance imaging has further characterised variations in subchondral plate thickness and its relationship to disc morphology and ligamentous attachments [3]. Pathological imaging studies, particularly in osteitis pubis, have also documented sclerosis, erosions, and cystic changes indicative of reactive remodelling under chronic mechanical stress or inflammation [14, 15].

Despite these contributions, existing studies of the pubic symphysis remain largely descriptive, are based on relatively small cohorts, and lack systematic quantitative validation of mineralisation patterns. In particular, no study to date has combined qualitative pattern classification with region-based quantitative HU analysis in a large cohort, nor examined bilateral pattern conformity or formally tested age-related effects on subchondral mineral density. Consequently, the extent to which previously reported sex differences can be generalised, quantified, and statistically validated remains unclear.

An important clinical correlate of pubic symphyseal biomechanics is groin pain, which is frequently associated with symphyseal pathology and shows sex-related differences in prevalence and presentation [14, 16]. Sex-specific pelvic morphology may lead to distinct loading regimes across the anterior pelvic ring, potentially explaining observed differences in mineralisation patterns as well as susceptibility to pain and degeneration [12, 17]. Addressing these gaps is therefore relevant not only from a biomechanical perspective but also for improving understanding of clinically significant symphyseal disorders.

Based on previous papers that account for sex-related differences in mineralisation and groin pain between males and females [12, 17], the following three hypotheses were investigated:

1. Pubic symphyseal surfaces exhibit sex-dependent mineralisation patterns.
2. Males show higher anterior mineralisation; females exhibit greater posterior mineralisation.
3. Age negatively correlates with bone mineral density.

Accordingly, the objective of this study was to systematically quantify and visualise subchondral bone mineral density distribution patterns of the pubic symphyseal surfaces using CT-OAM in a large cohort. Building upon earlier work, this study introduces a structured qualitative pattern classification, supplements it with region-specific quantitative HU analyses, and evaluates bilateral conformity and age-related effects. In doing so, it aims to provide a more comprehensive and statistically robust characterisation of pubic symphyseal subchondral adaptation under long-term physiological loading.

## Materials and methods

Eighty-five (51 males, 34 females; age range: 18–97 years, mean age: 65.3 ± 17.0 years) CT scans were used in this study, resulting in 170 individual pubic symphyseal surfaces analysed. Thirty scans (16 males, 14 females, age range; 18–82 years, mean age: 58.0 ± 17.1 years) were acquired from Dunedin Hospital. These were acquired for the diagnosis of non-musculoskeletal pathologies or to rule out injuries related to acute trauma. None of these cases had a current or past history of lower back or groin pain, sacroiliac joint-related pathology or abnormalities reported in previous medical records.

A further 55 pelvic CT scans (35 males, 20 females; age range: 39–97 years, mean age: 69.4 ± 15.6 years) were also analysed. The scans were acquired from individuals who donated their bodies to research at the Department of Anatomy, University of Basel. No apparent prior pelvic pathologies were identified radiologically upon inspection before the inclusion of the specimens for this study.

### CT-osteoabsorptiometry of the subchondral endplates

Data sets for CT-OAM were derived from conventional CT (Siemens Somatom S4, Siemens AG, Forchheim, Germany) from Basel and from Dunedin (scanner: SOMATOM as64 open, Siemens, Munich, Germany). Data sets for CT-OAM were derived from conventional CT (Siemens Somatom S4). Scanning parameters for the Basel cohort were as follows: convolution kernel- B31s (soft tissue filter), matrix- 512 × 512, field of view- 180 to 210 mm, voltage- 130 kV, slice thickness- 0.75 mm, exposure- 160-165mAs, voxel dimensions- 0.6 × 0.36 × 0.36. For the Dunedin cohort, parameters were: convolution kernel- B31s (soft tissue filter), matrix- 512 × 512, field of view- 500 mm, voltage- 120 kV, slice thickness- 1.25 mm, exposure time- 40-160mAs, voxel dimensions- 0.98 × 0.98 × 1.25.

CT-OAM was evaluated using ANALYZE (v11.0, Biomedical Imaging Resources, Mayo Foundation, Rochester, NY, USA). The left and right surfaces of the pubic symphysis were first manually segmented within the CT datasets to create 3D reconstructions of the individual hemipelves. These 3D models were then orientated into the optimal view of the pubic symphysis. Mineralisation data were then extracted from the manually isolated pubic symphysis surface and were then false color-coded and superimposed on the 3-dimensionally reconstructed pelvis for anatomical localization of the BMD. This creates a so-called colour densitogram as in prior studies [3, 18–23]. The maximum intensity projection revealed HU values to a depth of 3 mm, with thresholds set at ≤ 200 to ≥ 1200 HU.

### Qualitative pattern classification

**Fig. 1:**
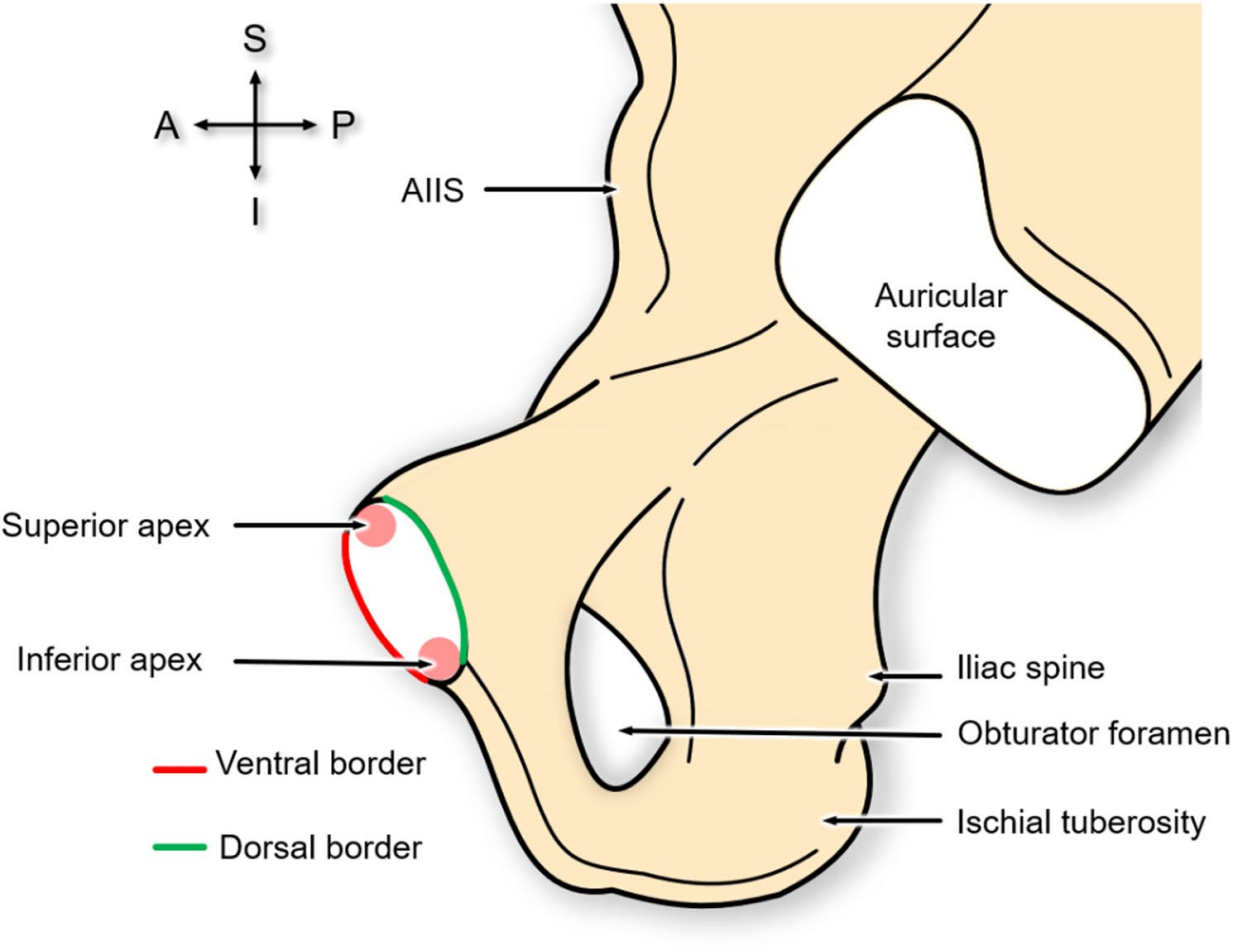
Medial view of a right innominate bone showcasing the pubic symphysis anatomy and terminology of its surface. A: anterior, I: inferior, AIIS: anterior inferior iliac spine, P: posterior, S: superior

The anatomy of the pubic symphysis is visualised in Fig. 1, where the areas of interest are highlighted as the apexes (superior and inferior) and the borders (ventral and dorsal).

**Fig. 2:**
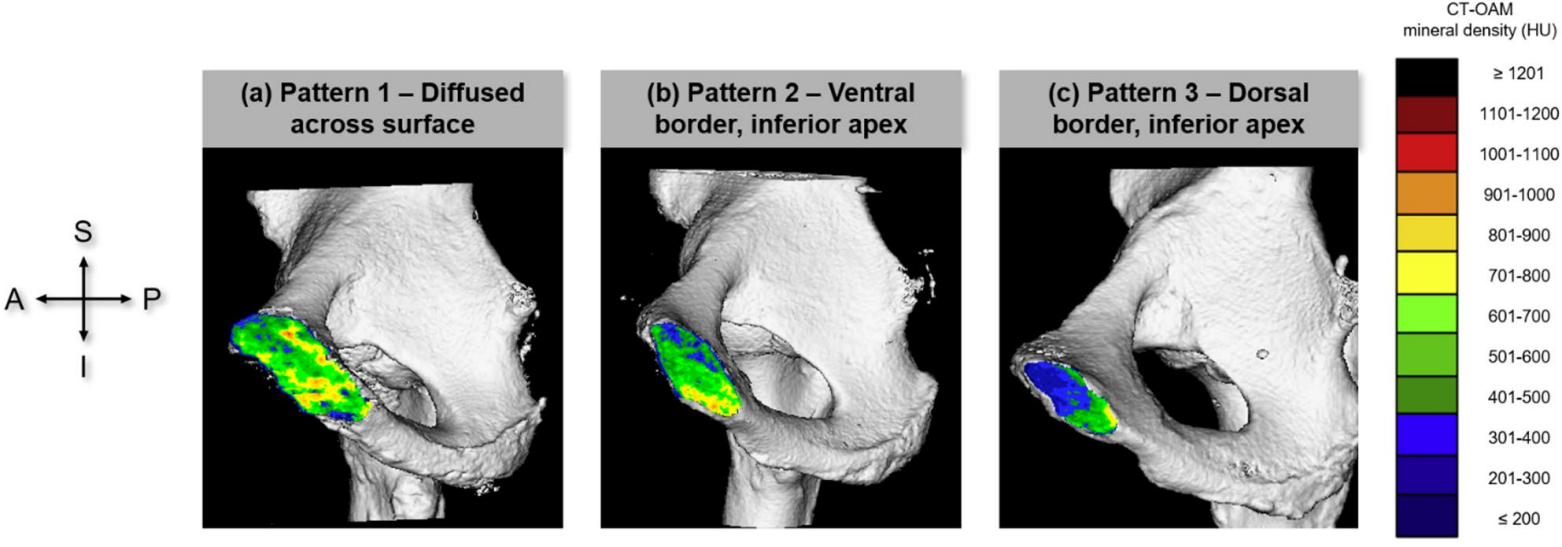
Pubic symphysis pattern classification into five main pattern groups. Highest mineralisation zones (see scale on the right) show three distinct patterns: (**a**) diffused, (**b**) ventral border and inferior apex, (**c**) dorsal border and inferior apex. A: anterior, I: inferior, P: posterior, S: superior

The assessment of mineral density patterns was made based on a semi-quantitative analysis of the entire surface region colour map of each joint surface. Based on the distribution of the highest mineralisation zones across the entire surface, the analysis revealed three main pattern types (Fig. 2):

- Pattern 1 presented a diffused mineralization across the surface with no specific maxima,
- Pattern 2 had highest mineralization located at ventral border with or without the inferior apex region,
- Pattern 3 had highest mineralization located at the dorsal border with or without the inferior apex region.

All of the specimens were categorised into each of the three pattern categories. The patterns of the contralateral sides of each specimen (left-right comparison) were compared to observe at whether the patterns were ‘conforming’ or ‘non-conforming’ as seen in previous studies [18, 24].

### Quantitative pattern classification

**Fig. 3:**
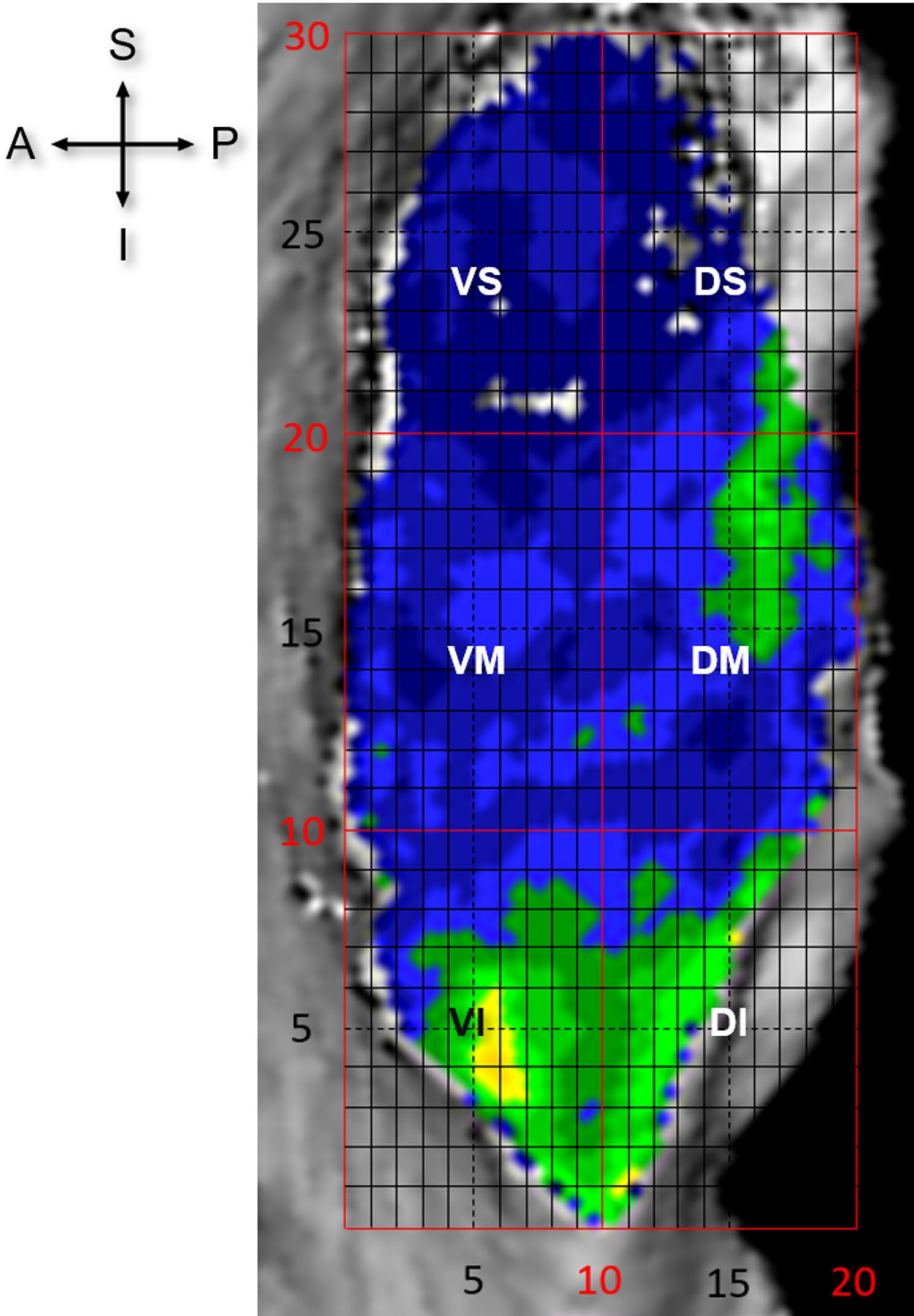
Grid analysis example using a 20 × 30 grid system positioned over the pubic symphysis in a sagittal plane to separate the six regions. A: anterior, DI: dorsal inferior, DM: dorsal middle, DS: dorsal superior, I: inferior, P: posterior, S: superior, VI: ventral inferior, VM: ventral middle, VS: ventral superior

BMD of the individual pubic symphyseal surfaces was assessed based on the mean HU values of the defined regions on the densitograms for each dataset as previously described for the sacroiliac joint [19]. The pubic symphysis surfaces were subdivided into six regions: ventral superior (VS), ventral middle (VM), ventral inferior (VI), dorsal superior (DS), dorsal middle (DM) and dorsal inferior (DI). These were defined as six sections of roughly equal size, created by a vertical line dividing the surface into ventral and dorsal halves and two horizontal lines equidistant from the superior and inferior apexes (Fig. 3). A grid tool was used to conform the shape of each specimen and create the six regions. Mean HU values for each region were calculated using non-calibrated CT grey values in ANALYZE, v11.0 using the ‘region of interest’ function. These values were subsequently statistically compared between the different groups.

GraphPad Prism (version 9, San Diego, CA, USA) was used for statistical analyses. Statistical significance was defined with a significance level of 0.05 (α < 0.05) and a 95% confidence interval. Gaussian distribution was first assessed using a Shapiro–Wilk test. Depending on the data distribution, a one-way ANOVA or a Kruskal–Wallis test with Dunn’s post-hoc correction was undertaken for the multiple assessment of the data between the six regions. Mean HU values were reported ± standard deviation. Age correlations with mean HU values in the three regions between sexes, sides and within the bone were assessed using a two-tailed Spearman r test for non-parametric data or a two-tailed Pearson r test for parametric data. Correlations were defined as follows: strong *r* ≥ 0.7, moderate 0.7 > *r* ≥ 0.5, weak 0.5 > *r* ≥ 0.2.

## Results

Population specific noise in the data between the two cohorts was compared, and no statistical differences were identified; therefore, pooled results are reported here. Cohort-specific results are reported in the supplementary materials.

### Pattern analyses-pubic symphyseal surfaces exhibit sex-dependent mineralisation patterns

**Fig. 4:**
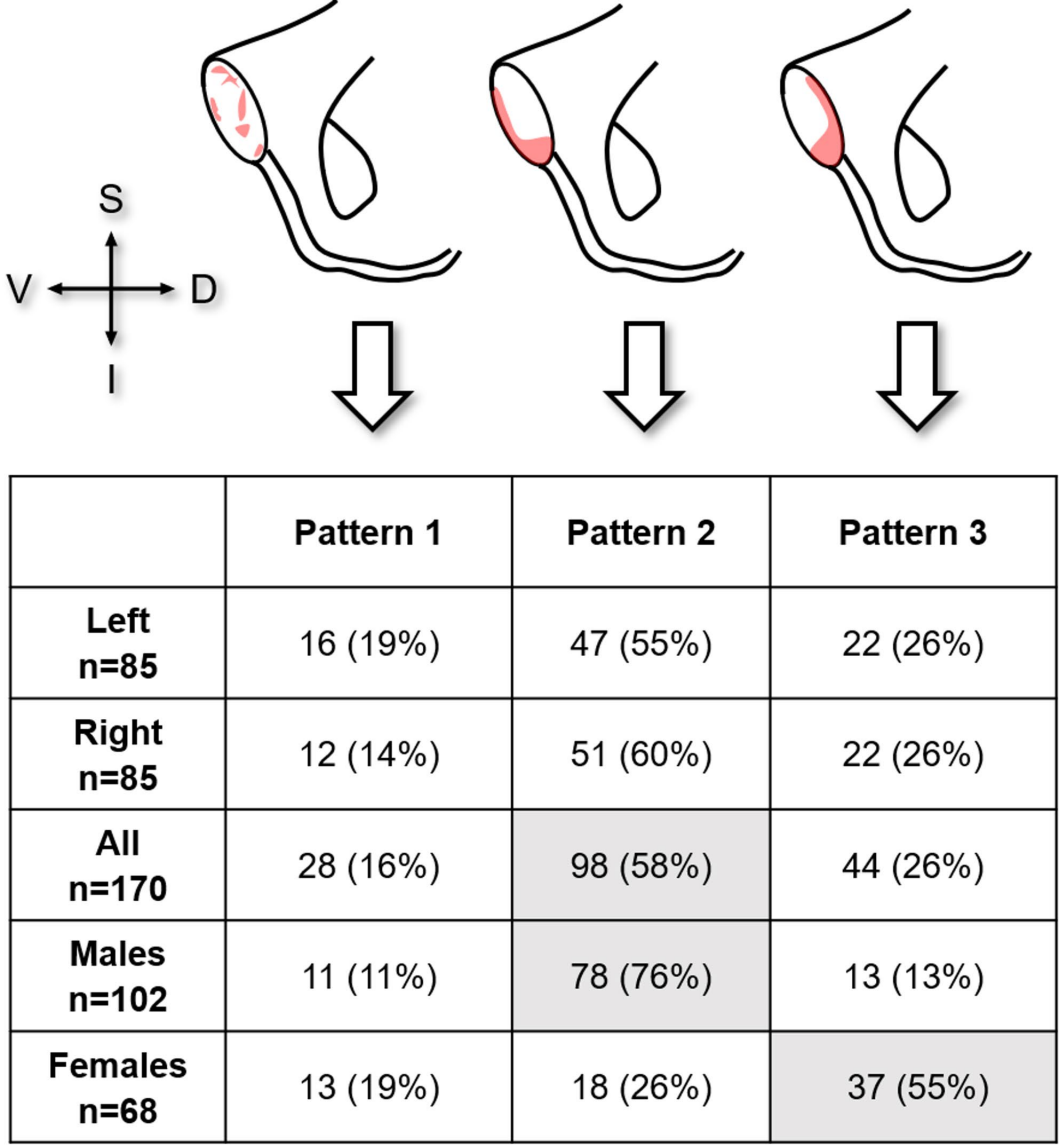
Distribution of patterns found in the cohort of pubic symphyses (*n* = 170 scans). D: dorsal, I: inferior, S: superior, V: ventral

Of the 170 surfaces analysed, the most common pattern found was pattern 2 (58%) when assessing at all pubic symphysis SCB. When stratified by sex, pattern 3 predominated in females (55%), whereas pattern 2 was most frequent in males (76%). The distribution of patterns found are presented in Fig. 4.

Conformity analyses of the patterns between corresponding left and right symphyses (*n* = 85) revealed that a majority of individuals exhibited bilaterally identical patterns of pubic symphysial SCB mineralisation (81%).

### Quantitative pattern classification: males show higher anterior mineralisation; females exhibit greater posterior mineralisation

**Fig. 5:**
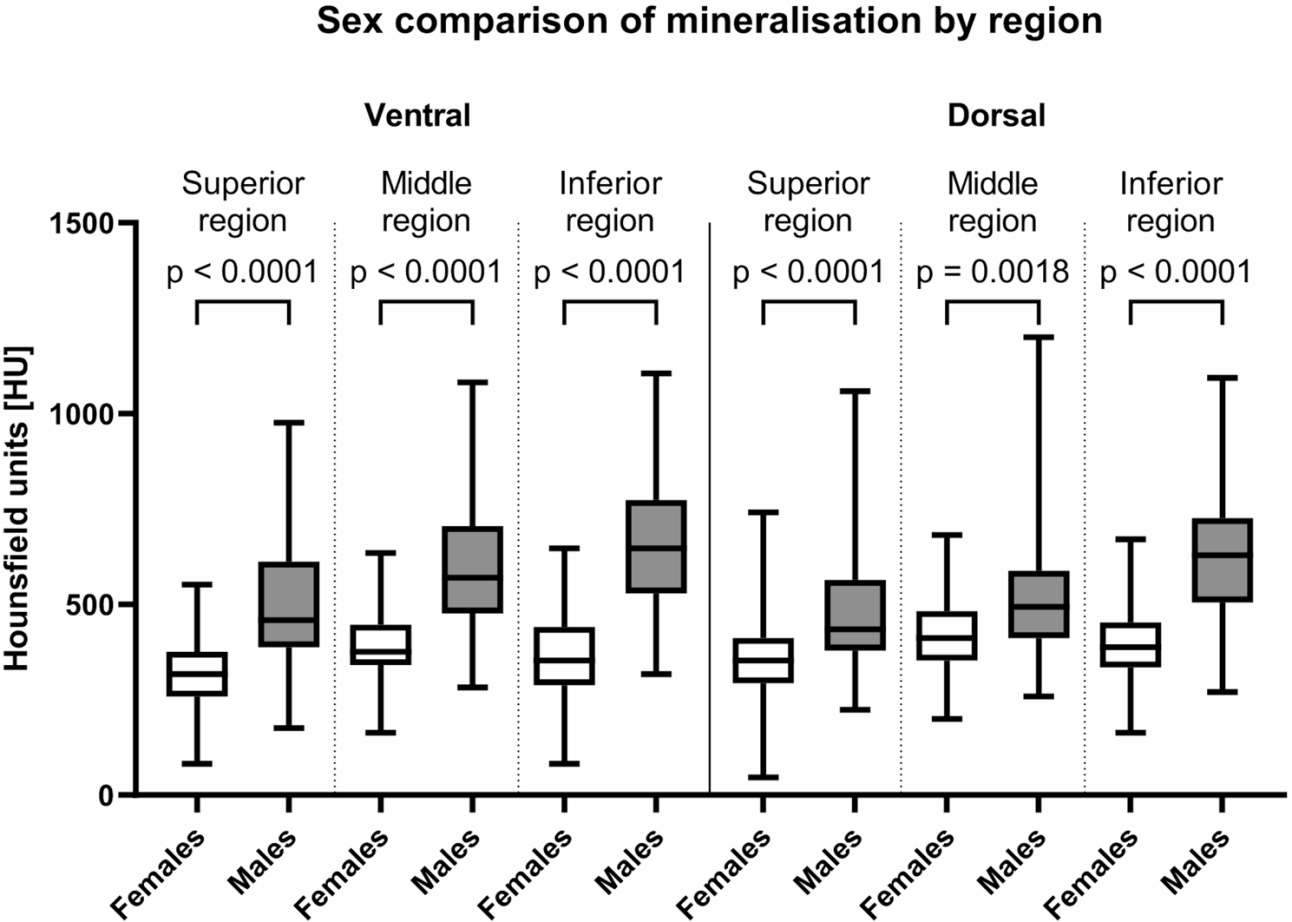
Distribution of mean Hounsfield Units of each region in males and females. Outlines of the boxes indicate the 25- and 75-percentile, the solid black horizontal line, the median. Whiskers indicate the 5–95 percentiles. The dotted lines separate the cohorts in the figure

Mean HU values of the pubic symphysis SCB of males and females were significantly lower in females than in males (females; 374 ± 111 HU; 95% CI: 363–385 HU, males; 554 ± 180 HU; 95% CI: 540–569 HU; *p* < 0.01). Males demonstrated statistically significantly higher HU values across all subregions compared with females (Fig. 5; Table 1). No significant differences were identified between left and right symphyseal surfaces (*p* > 0.9).

**Table 1:** Mean Hounsfield Unit (HU) values of each subregion with 95% confidence interval (CI) in males and females

	Ventral superior		Ventral middle		Ventral inferior	
	Mean (HU)	95% CI (HU)	Mean (HU)	95% CI (HU)	Mean (HU)	95% CI (HU)
Females	320 ± 99	296–343	390 ± 104	365–415	360 ± 106	335–386
Males	495 ± 164	462–528	598 ± 173	563–633	654 ± 170	619–688

	**Dorsal superior**		**Dorsal middle**		**Dorsal inferior**	
	Mean (HU)	95% CI (HU)	Mean (HU)	95% CI (HU)	Mean (HU)	95% CI (HU)
Females	360 ± 117	332–389	421 ± 110	395–418	393 ± 106	368–418
Males	475 ± 156	444–510	515 ± 155	483–546	629 ± 166	595–662

**Fig. 6:**
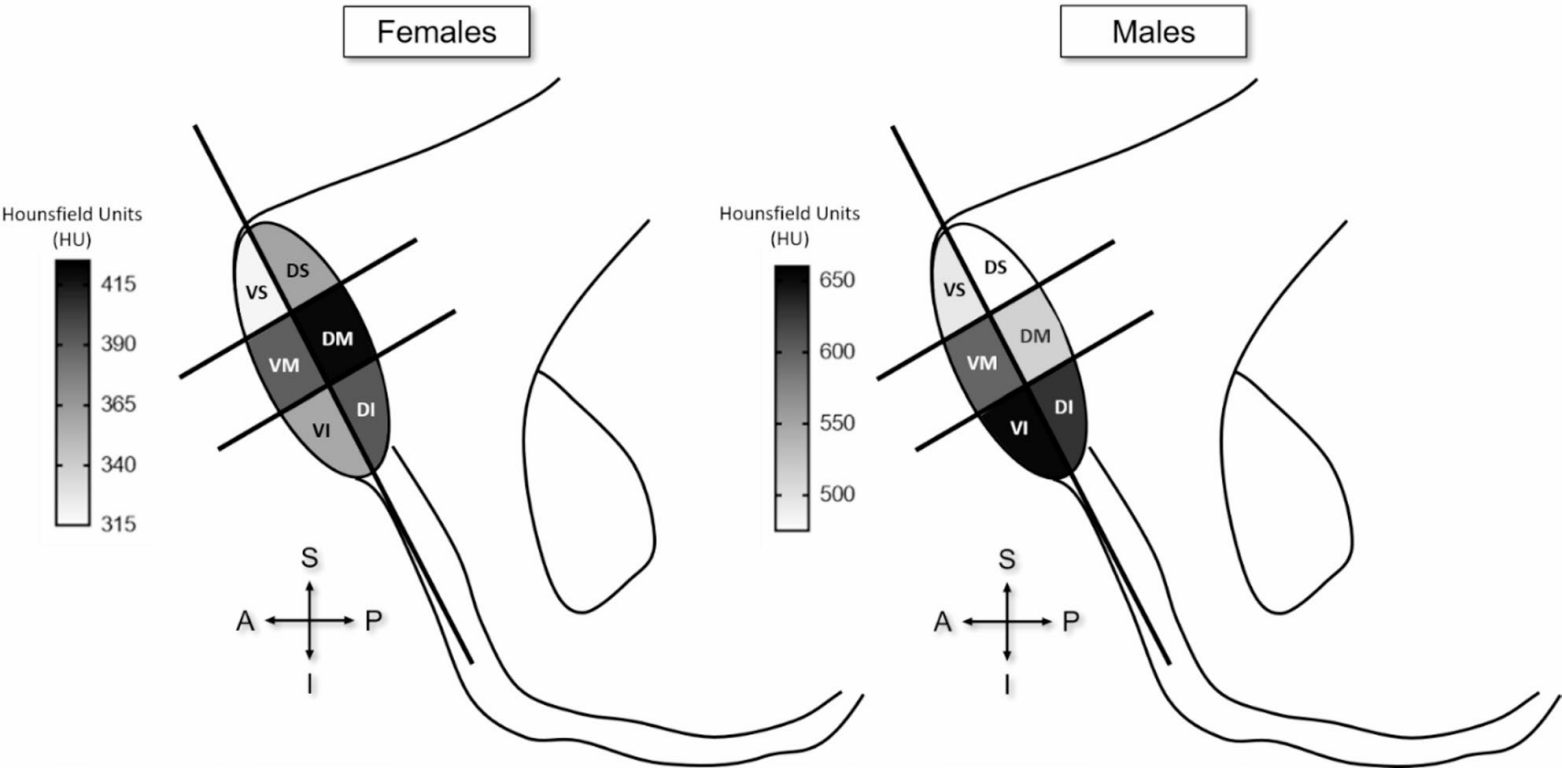
Distribution of mean Hounsfield Units of each region in males and females. DI: dorsal inferior, DM: dorsal middle, DS: dorsal superior, VI: ventral inferior, VM: ventral middle, VS: ventral superior

The mean HU value patterns are presented in Fig. 6 higlighting the sex-specific differences in mineralisation across the symphyseal SCB subregions. Patterns show higher mineralisation in the dorsal regions in females, whilst the ventral-inferior apex region is higher in males.

### Correlations with age- age negatively correlates with bone mineral density in the ventral middle region

**Fig. 7:**
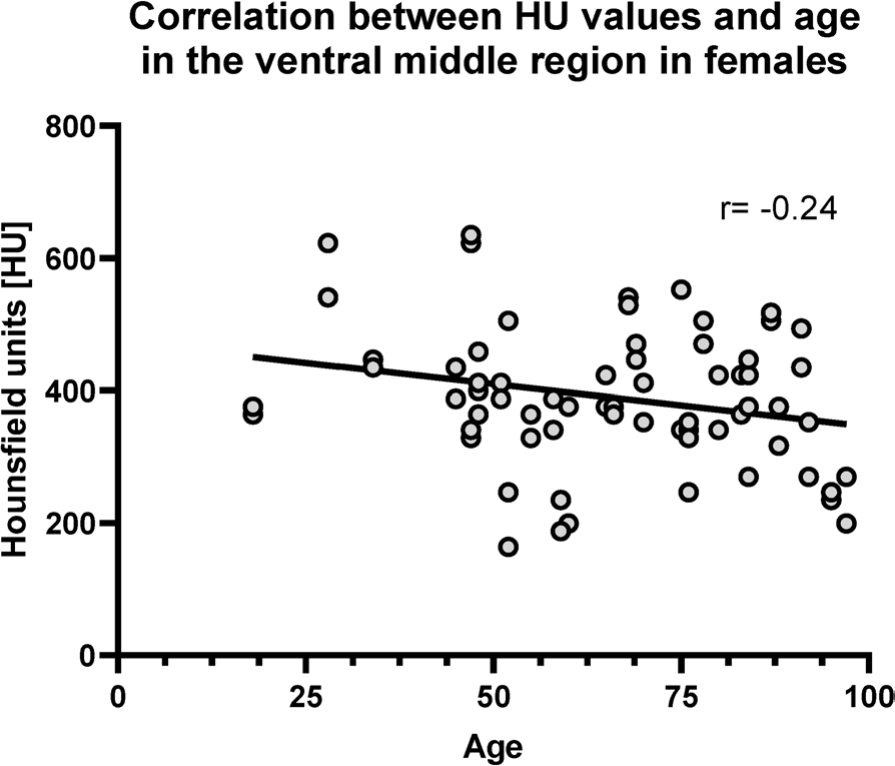
Correlation of mean HU values in the ventral middle region of the pubic symphyses in females

Correlations between age and mean HU values of the complete SCB did not differ between males (*p* = 0.34; *r* = 0.10) and females (*p* = 0.31; *r* = − 0.13). However, when looking at the specific subregions, age yielded a weak negative correlation in the ventral middle region in females (*p* < 0.05; *r* = -0.24) (Fig. 7). There were non the other subregions there were no statistically significant correlations between HU values and age (*p* > 0.46). In males, there were no statistically significant correlations between HU values and age in any of the regions (*p* > 0.1).

## Discussion

This study qualitatively analysed SCB mineralisation patterns of the pubic symphysial surfaces in a large cohort of patients with no known pelvic pain or dysfunction. It provides insights into the mechanical differences between individuals of different sexes and across a broad age range. Taken together, the observed mineralisation patterns are likely to reflect long-term, cumulative loading conditions acting on the pubic symphysis and thus offer a biomechanical signature of joint function.

Clear sex-dependent differences in subchondral mineralisation patterns were identified, confirming and extending earlier observations by Putz, et al. [12]. They state that males typically exhibit higher density anteriorly, while females show increased posterior density. This result was confirmed qualitatively in the present study, where pattern 3 (dorsal border and apex mineralisation) was the most common for females (55% of females). Pattern 2 (anterior border and apex mineralisation) was the most common occurring in males (76% of males). Furthermore, mineralisation patterns showed region-specific variations in SCB density across the surface dependent on sex. When subdividing the area into six subregions, mineral density distribution could be assessed based on the mean HU values which represent the mean mineralisation of that specific region. Highest mineralisation in females was localized in all dorsal regions as well as the ventral middle region, whilst in males it was concentrated antero-inferiorly in the ventral middle, ventral inferior and dorsal inferior regions. This qualitative patterning was reinforced by quantitative analyses, which demonstrated region-specific differences in mean HU values across the symphyseal surface. Notably, females displayed higher mineralisation in dorsal regions, while males showed greater mineralisation in the antero-inferior apex, indicating distinct loading regimes between the sexes. These findings directly support the first hypothesis, confirming that pubic symphyseal surfaces exhibit clear sex-dependent mineralisation patterns. They also support the second hypothesis, with males demonstrating predominantly anterior mineralisation while females showed relatively higher posterior mineralisation.

From a biomechanical perspective, these patterns are consistent with known differences in pelvic morphology and kinematics between males and females. The pubic symphysis is a cartilaginous, synarthrodial joint featuring a fibrocartilaginous interpubic disc and it allows only slight translational and rotational motion but is adapted to absorb both compressive and tensile stresses [11]. The pelvic ring is a closed-chain structure so any motion at the pubic symphysis must be accompanied by corresponding movement at the sacroiliac joint, and vice versa [25, 26]. The disc is wedged within the interpubic cavity between the two corresponding symphyseal surfaces and contains fibres of differing orientations. The upper and lower edges of the symphysis are reinforced mostly anteriorly by oblique running bundles of fibres, which can be viewed as solid bands. Due to the interweaving of the fibres within the interpubic disc, it also has the capacity to absorb vertical shear stresses in particular [12]. Hammer, et al. [25]’s kinematic study has described the movement of the innominate bones and pubic symphysis under physiological loading conditions. These are small movements, composed mainly of rotations in the vertical axis. The superior pubic ramus is rotated anteromedially relative to the ilium, and both pubic rami laterally, forming a counter movement effectively compressing the interpubic disc. However, the authors make no comment as to which specific areas of the pubic symphysis are more or less compressed within this movement owing to their experimental setup with surface deformations assessed exclusively [25]. Sex-related differences in pelvic kinematics have been documented, which may, in part, reflect the influence of the generally wider female pelvis [26, 27]. On average, females exhibit a modest anterior pelvic tilt of approximately 4° [26] whereas males typically maintain a pelvic orientation which is closer to neutral [28]. This difference could explain the stark differences in mineralisation of the symphysis in males and females found in this study. The anterior pelvic tilt in females may impact the symphysis in that the anterior part has a tendency to gape under chronic compressive loading which would in turn lower anterior mineralisation but heighten it posteriorly. The opposite would then be possible in males. In addition to pelvic morphology, sex-specific differences in muscle attachment may contribute to the observed mineralisation patterns. Jadzic, et al. [29] demonstrated that pubic bone muscle attachment sites are closely related to internal cortical and trabecular microarchitecture, with region- and sex-dependent adaptations. Increased anterior muscle loading in males and relatively greater posterior stabilising forces in females may therefore partly explain the observed anterior–posterior differences in subchondral mineralisation. These findings support a functional link between muscle-induced loading and region-specific subchondral adaptation at the pubic symphysis.

A key clinical implication of these structural and biomechanical characteristics is the occurrence of groin pain, which is often linked to pathology of the pubic symphysis such as osteitis pubis. The distinct pelvic morphologies of the sexes—most notably the broader female pelvis with its longer anterior lever arm—are likely to generate different loading conditions across the anterior pelvic ring. In females, this geometry may amplify bending moments at the pubic symphysis, leading to compressive stresses in the posterior (dorsal) region of the joint. In contrast, the narrower male pelvis may favor the development of higher shear forces at the symphysis. These sex-dependent loading patterns could account not only for observed differences in mineralisation but also for variations in the clinical presentation of groin pain. Furthermore, osteophyte formation, commonly seen in degenerative changes of the symphyseal surface, may serve as an indirect morphological indicator of these repetitive mechanical stresses [17].

Beyond qualitative patterning, males exhibited significantly higher overall subchondral bone mineralisation than females across all regions (374 HU in females vs. 554 HU in males). This was further apparent when separated by region. This finding suggests that males appear to have more chronic stress upon their joint than females. The pubic symphysis is subjected to various forces in daily chronic loading conditions, which include traction on the inferior part of the joint and compression of the superior region when standing, compression when sitting, and shearing and compression during single-leg stance [11, 30]. However, biomechanical differences between males and females remained poorly documented to date, and contemporary literature suggests that sex does not have an influence [30]. These results combined with the pattern analysis between males and females reflect the differences in chronic loading conditions that the pelvis is subjected to daily. Differences in pelvic morphology between males and females may stem from obstetric requirements, variations in growth trajectories, or a combination of both. However, there is no evidence supporting the idea that increased pelvic width affects locomotor efficiency in females [31]. The pubic symphysis is a region which supports a greater body weight load and is subject to more bone remodelling changes which is directly related to sex as males typically have higher body mass and greater muscle forces, increasing joint reaction forces transmitted through the pelvic ring and sacroiliac joint [32, 33] than females which would explain the significant difference in mineralisation between the sexes. Additionally, childbirth may further shape symphyseal biomechanics in females: pregnancy-related hormonal changes induce ligamentous laxity and widening of the symphysis, while vaginal delivery imposes substantial transient deformation and strain on the joint. These cyclical or cumulative stresses have been associated with alterations in subchondral bone architecture, postpartum remodeling, and, in some cases, degenerative changes of the symphyseal margins [34]. As a result, parity may contribute to the lower overall mineralization observed in females, although current evidence remains limited and heterogeneous.

In contrast to sex-related differences, age showed only a limited influence on subchondral mineralisation. Although a weak negative correlation was identified in the ventral middle region in females, no consistent age-related decline in mineralisation was observed across regions or in males. Previous studies have reported significant correlations with biological age resulting from bone density and chronological age [32, 35]. These reports however, specifically looked into pubic cancellous bone and excluded the subchondral bone plate. No other study has investigated the SCB of the pubic symphysis and correlated it with age as the given study does so these results are novel. It was expected, based on the literature that there would be evidence of a negative correlation of BMD with age especially in females due to the numerous morphological changes that may occur (hormonal factors, osteoporosis or hormone replacement treatment). The results show evidence of weak correlations (*r* < − 0.2) but these findings were not significant. Accordingly, the third hypothesis was not supported, suggesting that subchondral adaptation at the pubic symphysis may be maintained across adulthood under physiological loading conditions.

It should be acknowledged that CT-osteoabsorptiometry provides an indirect, macroscopic assessment of subchondral mineralisation and cannot replace histological or micromorphological analyses. Histological techniques would allow direct evaluation of tissue-level mineralisation, microdamage, and remodelling dynamics and would therefore represent an important complementary approach. However, such analyses are not feasible retrospectively in the absence of harvested tissue. Importantly, this limitation does not diminish the value of the present findings; rather, it highlights the role of CT-based methods as a non-destructive tool capable of capturing spatial mineralisation patterns in large cohorts. While CT imaging is inherently limited compared with histological sections in terms of microstructural resolution, it offers a unique advantage in assessing joint-level adaptations under physiological loading in vivo. Future studies combining CT-OAM with histology or high-resolution bone imaging techniques would therefore represent a valuable next step to further elucidate the structural basis of the observed mineralisation patterns.

Several limitations of this study should be acknowledged. The cross-sectional study design limits interpretation to a single time point and precludes assessment of longitudinal changes or progression of subchondral bone mineralisation with age. The analysed material comprised two distinct cohorts—clinical CT scans from living individuals and post-mortem scans from donated specimens. While post-mortem scans were acquired within hours of death and were unlikely to be influenced by decomposition, the combination of these cohorts may introduce selection bias. Furthermore, detailed demographic and clinical information, including ethnicity, comorbidities, pharmacotherapies, parity status, physical activity, alcohol consumption, and smoking history, was unavailable and could not be accounted for, representing potential sources of unmeasured confounding. No a priori sample size or power calculation was performed due to the retrospective, exploratory design of the study. While a post hoc power analysis could have been conducted to further contextualise non-significant findings, this was not included and therefore represents a limitation of the present work. CT-osteoabsorptiometry (CT-OAM) is not a standardised method for absolute bone mineral density assessment. Unlike quantitative backscattered electron imaging (qBEI), which directly measures tissue-level mineralisation [36, 37], CT-OAM provides a relative assessment based on Hounsfield Units that are influenced by scanner settings and reconstruction parameters [38]. Consequently, CT-OAM reflects relative rather than absolute mineral density.

Histology and micro-computed tomography (micro-CT) allow direct evaluation of tissue-level mineralisation and microarchitecture but are limited in humans by the trade-off between spatial resolution and field of view required to maintain acceptable radiation doses. Although in vivo micro-CT has been demonstrated experimentally, it is not currently feasible for assessment of the human pubic symphysis [39]. Previous micro-CT studies, such as Jadzic, et al. [29] characterised subchondral cortical and trabecular microarchitecture of the pubic symphysis in relation to age and sex. While micro-CT resolves fine microstructural features beyond the capability of clinical CT, both techniques address complementary aspects of bone adaptation. Micro-CT captures tissue-level architecture in small ex vivo samples, whereas CT-OAM enables non-destructive, surface-based mapping of mineralisation patterns across the entire joint in large cohorts. Accordingly, the present study extends existing work by providing a macroscopic, functionally oriented assessment of joint-level loading adaptation. Micro-CT was not feasible here because part of the cohort comprised living subjects.

Finally, the grid placement and regional segmentation were performed by a single investigator, and inter-observer reproducibility was not assessed. Although qualitative pattern classification was performed with agreement between two authors, this approach remains partially subjective and warrants further validation in future studies.

## Conclusion

CT-osteoabsorptiometry of the pubic symphysis reveals sex- and region-specific mineralisation patterns that reflect long-term biomechanical loading. These adaptations help explain differences in susceptibility to groin pain and degenerative changes. By linking subchondral mineral density to pelvic morphology and clinical presentation, this study establishes CT-OAM as a valuable tool for investigating pubic symphyseal biomechanics and provides a basis for future functional and pathological research.

## Supplementary Information

Supplementary Material 1.

## Data Availability

The data acquired in the course of this study are available from the corresponding author on request.

## Abbreviations

BMD: Bone mineral density
CI: Confidence interval
CT-OAM: Computed tomography osteoabsorptiometry
DI: Dorsal inferior
DM: Dorsal middle
DS: Dorsal superior
HU: Hounsfield Units
SCB: Subchondral bone
VI: Ventral inferior
VM: Ventral middle
VS: Ventral superior
