# Supplementary material for: Sex and age differences in regional distribution of bone mineral density in the pubic symphysis using Computed Tomography Osteoabsorptiometry (CT-OAM)
Source: BMC Musculoskelet Disord. 2026 Feb 7;27:207. doi: 10.1186/s12891-026-09566-7 (PMC12977925; doi:10.1186/s12891-026-09566-7)
Supplement: Supplementary file 1 — Supplementary Material 1. [file 12891_2026_9566_MOESM1_ESM.pdf]

Group A- Basel

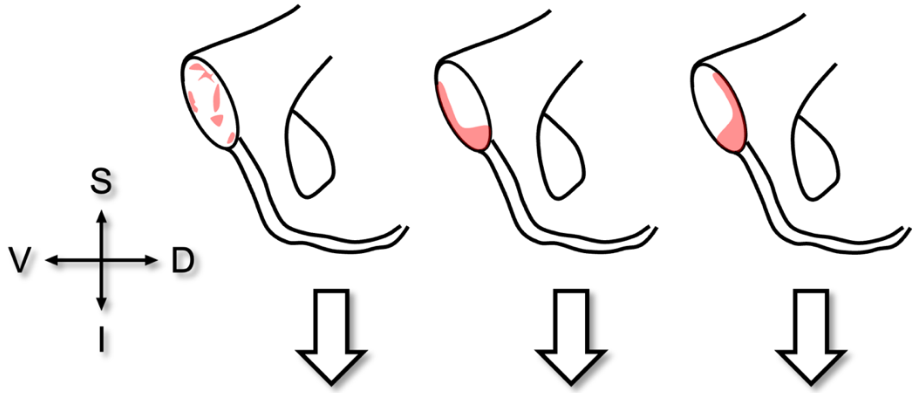

	Pattern 1	Pattern 2	Pattern 3
**Left n=55**	9 (16%)	31 (57%)	15 (27%)
**Right n=55**	7 (13%)	33 (60%)	15 (27%)
**All n=110**	16 (15%)	64 (58%)	30 (27%)
**Males n=70**	7 (10%)	53 (76%)	10 (14%)
**Females n=40**	9 (23%)	11 (27%)	20 (50%)

Supplementary figure 1: *Distribution of patterns found in the cohort of pubic symphyses from Basel (n=110 scans). D: dorsal, I: inferior, S: superior, V: ventral*

	Ventral superior		Ventral middle		Ventral inferior	
	Mean (HU)	95% CI (HU)	Mean (HU)	95% CI (HU)	Mean (HU)	95% CI (HU)
**Females**	327 ± 95	297-357	363 ± 86	335-390	347 ± 120	308-385
**Males**	483 ± 172	442-524	585 ± 172	544-626	650 ± 173	609-692
	**Dorsal superior**		**Dorsal middle**		**Dorsal inferior**	
	Mean (HU)	95% CI (HU)	Mean (HU)	95% CI (HU)	Mean (HU)	95% CI (HU)
**Females**	360 ± 131	318-401	410 ± 110	374-445	380 ± 109	346-415
**Males**	466 ± 161	428-504	501 ± 164	462-540	614 ± 163	575-653

Table 1: *Median Hounsfield Unit (HU) values of each subregion with range (minimum-maximum) in males and females from the Basel cohort.*

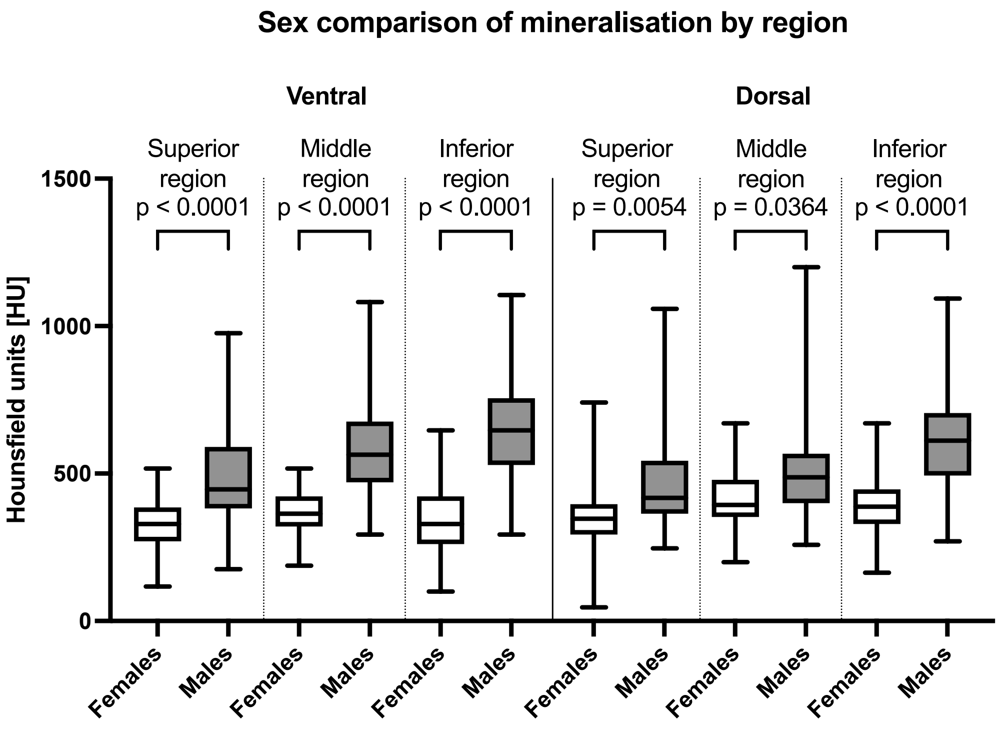

Supplementary figure 2: *Distribution of Hounsfield Units of each region in males and females in the Basel cohort of specimens. Outlines of the boxes indicate the 25- and 75-percentile, the solid black horizontal line, the median. Whiskers indicate the 5-95 percentiles. Dotted lines separate cohorts.*

Group B- Dunedin

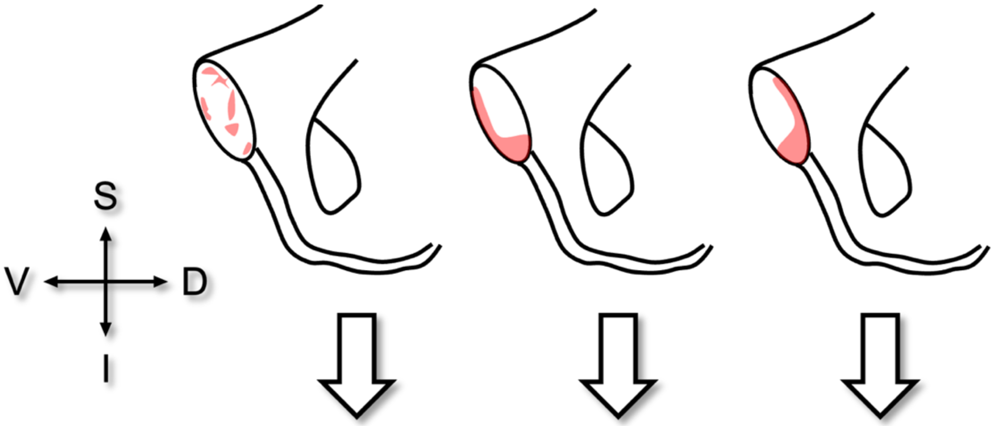

	Pattern 1	Pattern 2	Pattern 3
**Left n=30**	7 (23%)	16 (54%)	7 (23%)
**Right n=30**	5 (17%)	18 (60%)	7 (23%)
**All n=60**	12 (20%)	34 (57%)	14 (23%)
**Males n=32**	4 (13%)	25 (78%)	3 (9%)
**Females n=28**	8 (29%)	9 (32%)	11 (39%)

Supplementary figure 3: *Distribution of patterns found in the cohort of pubic symphyses from Dunedin (n=60 scans). D: dorsal, I: inferior, S: superior, V: ventral*

	Ventral superior		Ventral middle		Ventral inferior	
	Mean (HU)	95% CI (HU)	Mean (HU)	95% CI (HU)	Mean (HU)	95% CI (HU)
**Females**	309 ± 107	268-351	429± 118	384-475	375 ± 98	337-413
**Males**	520 ± 146	468-573	622 ± 184	556-688	644 ± 179	580-708
	**Dorsal superior**		**Dorsal middle**		**Dorsal inferior**	
	Mean (HU)	95% CI (HU)	Mean (HU)	95% CI (HU)	Mean (HU)	95% CI (HU)
**Females**	361 ± 99	323-400	437 ± 110	394-371	411 ± 103	371-451
**Males**	492 ± 146	440-545	547 ± 143	495-598	650± 176	586-713

Table 2: *Median Hounsfield Unit (HU) values of each subregion with range (minimum-maximum) in males and females from the Dunedin cohort.*

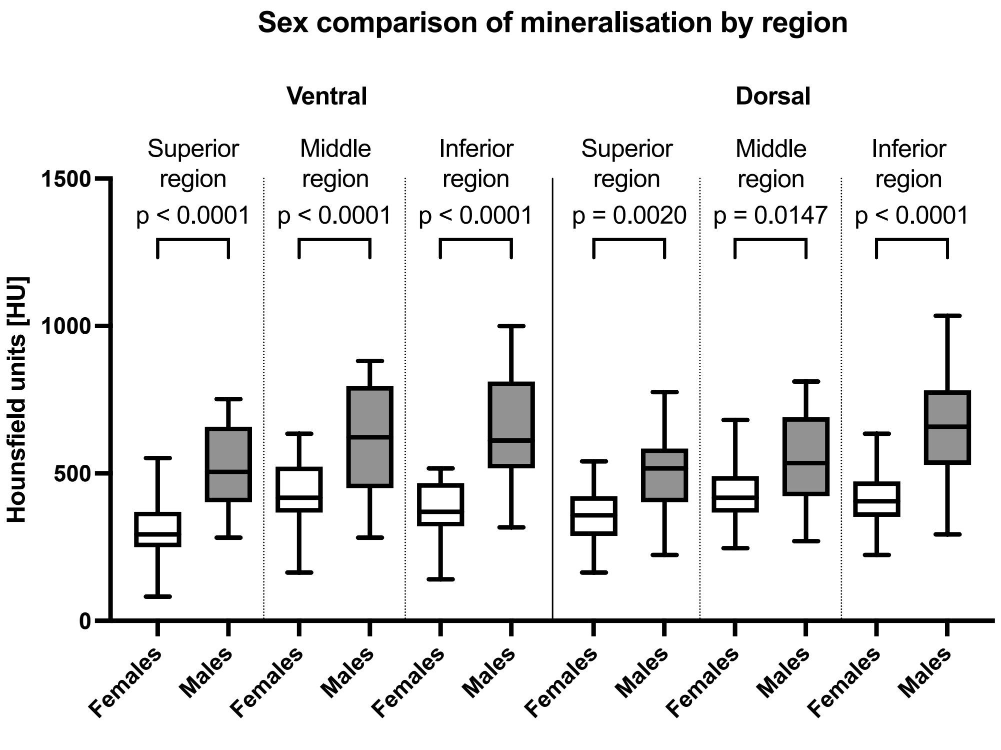

Supplementary figure 4: *Distribution of Hounsfield Units of each region in males and females* in the Dunedin cohort of specimens. *Outlines of the boxes indicate the 25- and 75-percentile, the solid black horizontal line, the median. Whiskers indicate the 5-95 percentiles. Dotted lines separate cohorts.*
